# Hyperbolic matrix factorization improves prediction of drug-target associations

**DOI:** 10.1038/s41598-023-27995-5

**Published:** 2023-01-18

**Authors:** Aleksandar Poleksic

**Affiliations:** Department of Computer Science, University of Northern Iowa, Cedar Falls, IA 50614 USA

**Keywords:** Machine learning, Virtual drug screening, Systems biology, Applied mathematics

## Abstract

Past research in computational systems biology has focused more on the development and applications of advanced statistical and numerical optimization techniques and much less on understanding the geometry of the biological space. By representing biological entities as points in a low dimensional Euclidean space, state-of-the-art methods for drug-target interaction (DTI) prediction implicitly assume the flat geometry of the biological space. In contrast, recent theoretical studies suggest that biological systems exhibit tree-like topology with a high degree of clustering. As a consequence, embedding a biological system in a flat space leads to distortion of distances between biological objects. Here, we present a novel matrix factorization methodology for drug-target interaction prediction that uses hyperbolic space as the latent biological space. When benchmarked against classical, Euclidean methods, hyperbolic matrix factorization exhibits superior accuracy while lowering embedding dimension by an order of magnitude. We see this as additional evidence that the hyperbolic geometry underpins large biological networks.

## Introduction

Computational methods for biological relationship inference use dimension reduction techniques to represent biological objects as points in a low-dimensional space. The underlying assumption is that biological systems have low intrinsic dimension. For instance, it has been well established that most variations in genomic databases can be explained by a small set of features, such as the cell state, the cell type, or a gene program^1^. In a different example, the low dimensionality of databases of drugs’ adverse reactions is due to associations of side-effects to chemical substructures and their combinations^2,3^. To put it differently, it is known that drugs sharing chemical substructures give rise to same adverse reactions.

**Figure 1:**
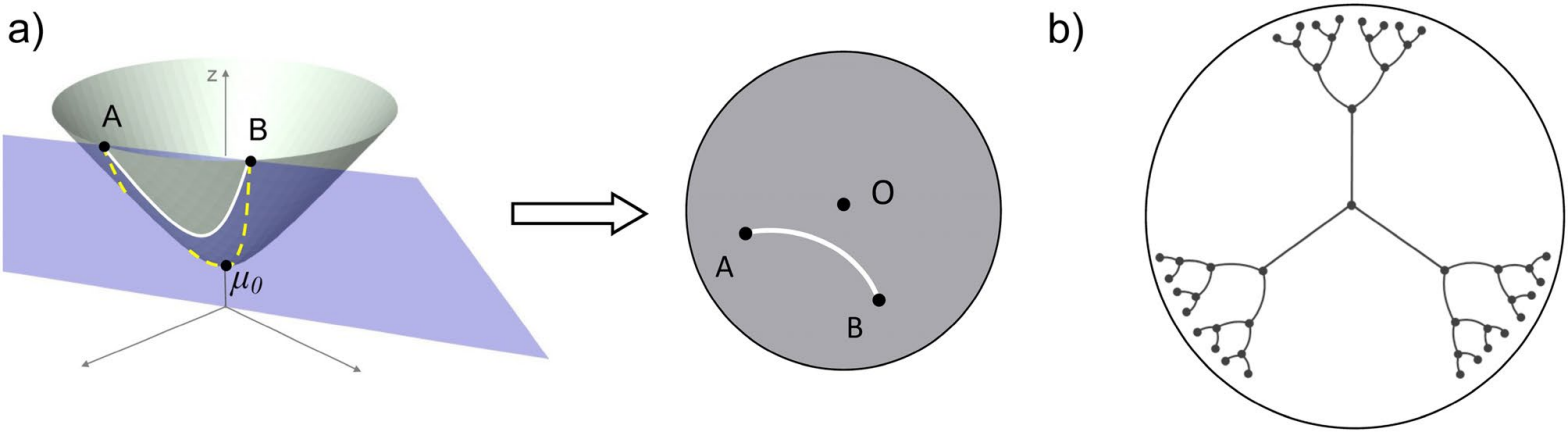
(**a**) Left: Hyperboloid model of ${\mathbb{H}}^{2}$. The shortest path between points A and B is the line of intersection of the hyperboloid with the plane (blue) that passes through A, B and O. As A and B are moving away from the vertex ${\mu }_{0}$, the length of this geodesic line (white) is almost the same as the length of the path through ${\mu }_{0}$ (orange). Right: Projection onto the Poincare disk. (**b**) Effective embedding of a tree into the hyperbolic space (all tree edges are of the same length).

The research on dimensionality reduction and associated relationship prediction has traditionally focused on the development and applications of advanced computational and statistical techniques while taking the Euclidean geometry of the native biological space for granted. However, recent theoretical studies challenge the flat geometry assumption^4–10^. According to these studies, complex systems exhibit tree-like topology with high degree of clustering. Therefore, embedding those systems into the Euclidean space inevitably leads to distortion of distances between individual objects and, in turn, compromises the accuracy of relationship inference. In contrast, a negatively curved space can accommodate the exponential growth in the number of relevant network features since the area of a hyperbolic circle is an exponential function of its radius (Fig. 1).

Recent years have seen the development of practical algorithms that use hyperbolic geometry to model complex networks^11–18^. Papadopoulos et al*.* developed the HyperMap method for mapping a complex network into a hyperbolic space^5^. Muscoloni et al*.* address the same problem using a technique based on the angular coalescence principle^14^. Monath et al*.* use a representation of tree structures in the Poincaré ball to design more accurate hierarchical clustering methods^15^. Mirvakhabova et al. propose a hyperbolic autoencoder algorithm for the classical collaborative filtering task^16^. Vinh Tran et al*.* propose a novel way of exploring metric learning for recommender systems in a hyperbolic space^17^. Schmeier et al*.* use Poincaré embeddings of hierarchical entities to develop and prioritize playlists for users of digital music services^18^. Hyperbolic distance learning has also been incorporated into artificial neural network models, for instance to encode the chemical structures of drugs^19^.

In this paper, we show how hyperbolic latent space can be utilized to increase the accuracy of matrix factorization. While our algorithm has been benchmarked on drug-target interaction datasets, the same technique can be applied to other relationship inference tasks (e.g., to predict drug-disease or drug-side effect associations, user preferences to movies or songs, etc.).

We emphasize that improving matrix factorization techniques is of particular importance in recommender systems, since a carefully designed matrix factorization method is known to outperform deep learning in many collaborative filtering applications. Specifically, while deep learning can theoretically optimize any function, learning a simple Euclidean dot product (employed in matrix factorization) is shown to be a non-trivial task^20^.

We incorporated hyperbolic latent space representation into the logistic matrix factorization framework, which is widely used in drug-target association prediction methods. We demonstrate that using the hyperbolic distance in place of the Euclidean distance results in significant accuracy improvements, while lowering the latent space dimension by more than an order of magnitude.

The rest of this article is organized as follows. "The theoretical foundation" section provides a short introduction into the hyperbolic geometry. In “Computing the prior distribution” and "The loss function" sections, we derive a hyperbolic variant of the logistic loss function used in several state-of-the-art matrix factorization method^21–24^. "Alternating gradient descent in hyperbolic space" section describes an alternating gradient descent procedure for minimizing the loss function. In “Hyperbolic neighborhood regularization and cold-start” section, we develop the hyperbolic versions of the neighborhood regularization and cold-start procedures. Finally, in the Results section we discuss the accuracy of hyperbolic and Euclidean matrix factorization algorithms on some widely used drug-target interaction test sets.

## Methods

### The theoretical foundation

Hyperbolic geometry can be modeled on the $n$-dimensional hyperboloid in the Lorentzian space ${\mathbb{R}}^{n,1}$ (Fig. 1), where ${\mathbb{R}}^{n,1}$ is a copy of ${\mathbb{R}}^{n+1}$ equipped with a bilinear form ${\langle \cdot ,\cdot \rangle }_{\mathcal{L}}$ defined as

1 $${\langle x,y\rangle }_{\mathcal{L}}={x}_{1}{y}_{1}+\dots +{x}_{n}{y}_{n}-{x}_{n+1}{y}_{n+1}.$$

Hyperbolic space is represented by one sheet of the two-sheeted hyperboloid

2 $$\left\{x\in {\mathbb{R}}^{n,1}|{\langle x,x\rangle }_{\mathcal{L}} =-1\right\}$$

(which can be thought of as a sphere of radius $i=\sqrt{-1}$ ), namely,

3 $${\mathbb{H}}^{n}=\left\{x\in {\mathbb{R}}^{n,1}|{\langle x,x\rangle }_{\mathcal{L}} =-1, {x}_{n+1}>0\right\}.$$

It can be shown that the bilinear form ${\langle \cdot ,\cdot \rangle }_{\mathcal{L}}$ restricted on the tangent space ${T}_{p}{\mathbb{H}}^{n}$ at a point $p\in {\mathbb{H}}^{n}$, defined by

4 $${T}_{p}{\mathbb{H}}^{n}=\left\{x\in {\mathbb{R}}^{n,1}|{\langle p,x\rangle }_{\mathcal{L}}=0\right\}.$$

is positive definite, thereby providing a genuine Riemannian metric on ${\mathbb{H}}^{n}$. The distance between two points $x$, $y\in {\mathbb{H}}^{n}$ is given by

5 $${d}_{{\mathbb{H}}^{n}}\left(x,y\right)=\mathrm{arccosh}\left({-\langle x,y\rangle }_{\mathcal{L}}\right).$$

An interesting (and in the biological context insightful) property of the hyperbolic space is that the shortest path between two random points in ${\mathbb{H}}^{{\varvec{n}}}$ that are far away from the vertex ${\mu }_{0}$ has almost the same length as the path through the vertex (Fig. 1). This resembles the property of the distance function on trees, where the shortest path between two randomly selected nodes deep in the tree is almost of the same length as the path through the root.

While the hyperbolic matrix factorization, outlined below, is applicable to different loss functions, we illustrate it in the framework of logistic matrix factorization. Logistic factorization technique is statistically sound, simple to present, and highly accurate in biological applications^21–28^.

Let $A={\left\{{a}^{i}\right\}}_{i=1}^{m}$ be the set of drugs and $B={\left\{{b}^{j}\right\}}_{j=1}^{n}$ the set of targets (proteins). Denote by $R={\left({r}_{i,j}\right)}_{m\times n}$ the matrix of relationships (edges) between the elements of $A$ and $B$. Specifically, ${r}_{i,j}=1$ if ${a}^{i}$ interacts with ${b}^{j}$ and ${r}_{i,j}=0$ otherwise (no interaction or unknown). Let ${u}^{i}$, ${v}^{j}\in {\mathbb{H}}^{d}$ be the latent vector representations of ${a}^{i}$ and ${b}^{j}$, respectively, where $d\ll \mathrm{max}\left(m,n\right)$. Denote by ${e}_{i,j}$ the event that ${a}^{i}$ interacts with ${b}^{j}$. In line with the classical (Euclidean) logistic matrix factorization technique^21–33^, we model the probability ${p}_{ij}$ of ${e}_{i,j}$ as the logistic function in the Lorentz space ${\mathbb{R}}^{d,1}$

6 $${p}_{i,j}=p\left({r}_{i,j}=1|{u}^{i},{v}^{j}\right)=\frac{\mathrm{exp}\left(-{d}_{\mathcal{L}}^{2}\left({u}^{i},{v}^{j}\right)\right)}{1+\mathrm{exp}\left(-{d}_{\mathcal{L}}^{2}\left({u}^{i},{v}^{j}\right)\right)},$$

where ${d}_{\mathcal{L}}^{2}\left(x,y\right)$ denotes the squared *Lorentzian distance*^34^ between the points $x,y\in {\mathbb{H}}^{d}$, namely

7 $${d}_{\mathcal{L}}^{2}\left(x,y\right)={\Vert x-y\Vert }_{\mathcal{L}}^{2}={\langle x-y,x-y\rangle }_{\mathcal{L}}=-2-2{\langle x,y\rangle }_{\mathcal{L}}.$$

Denote by $W={\left({w}_{i,j}\right)}_{m\times n}$ our confidence in the entries ${r}_{i,j}$ of the interaction matrix $R$. In many practical applications, ${w}_{i,j}=1$ if ${r}_{i,j}=0$, and ${w}_{i,j}=c$ if ${r}_{i,j}=1$, where $c>1$ is a constant^21^. In general, the idea is to assign higher weights to trustworthy pairs i.e., those for which we have higher confidence of interaction. Given the weights ${w}_{i,j}$, the likelihood of ${r}_{i,j}$ given ${u}^{i}$ and ${v}^{j}$ is

8 $$p\left({r}_{i,j}|{u}^{i},{v}^{j}\right)={p}_{i,j}^{{w}_{i,j}{r}_{i,j}}{\left(1-{p}_{i,j}\right)}^{{w}_{i,j}\left(1-{r}_{i,j}\right)}.$$

Thus, assuming the independence of events ${e}_{i,j}$, it follows that

9 $$p\left(R|U,V\right)=\prod_{i,j}{\left({p}_{i,j}^{{r}_{i,j}}{\left(1-{p}_{i,j}\right)}^{1-{r}_{i,j}}\right)}^{{w}_{i,j}},$$

where $U$ and $V$ represent the matrices of latent preferences of elements from $A$ and $B$, respectively (in other words, the ${i}$th row of $U$ is the vector ${u}^{i}$ and ${i}$th row of $V$ is ${v}^{i}$).

### Computing the prior distribution

Similar to the Euclidean case^21,31^, our goal is to derive the probability $p\left(U,V|R\right)$ from (9) through the Bayesian inference.

Utilizing the recent work on wrapped normal distribution in hyperbolic space^35^, we define the prior distributions as

10 $$\begin{aligned} p\left(U|{\upsigma }_{U}^{2}\right) & =\prod \limits_{i=1}^{m}\mathcal{G}\left({u}_{i}|{\mu }_{0},{\upsigma }_{U}^{2}I\right), \\ p\left(V|{\sigma }_{V}^{2}\right) & = \prod \limits_{j=1}^{n}\mathcal{G}\left({v}_{j}|{\mu }_{0},{\sigma }_{V}^{2}I\right),\end{aligned}$$

where $\mathcal{G}\left(\mu ,\Sigma \right)$ is the pseudo-hyperbolic Gaussian distribution and ${\mu }_{0}=\left(0,\dots ,\mathrm{0,1}\right)$ is the vertex of the hyperboloid (the origin of the hyperbolic space).

**Figure 2:**
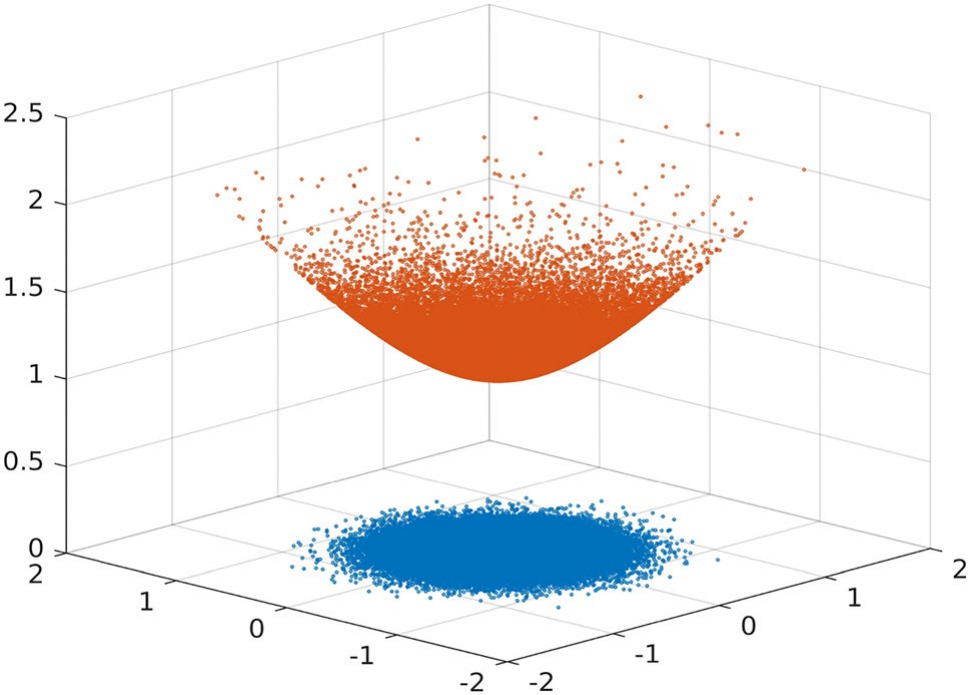
100,000 samples from $\mathcal{N}\left(0,\Sigma \right)$ (blue) and the corresponding samples from $\mathcal{G}({\mu }_{0},\Sigma )$ (red), $\Sigma =0.1 \cdot {I}_{2\times 2}$.

The pseudo-hyperbolic Gaussian distribution extends the notion of Gaussian distribution to the hyperbolic space (Fig. 2). In short, for $\mu \in {\mathbb{H}}^{d}$ and positive definite $\Sigma$, sampling from $\mathcal{G}\left(\mu ,\Sigma \right)$ can be thought of as a three step process: (a) Sample a vector $x\in {T}_{{\mu }_{0}}{\mathbb{H}}^{d}$ from $\mathcal{N}\left(0,\Sigma \right)$, (b) Transport $x$ along the geodesic joining the points ${\mu }_{0}\in {\mathbb{H}}^{d}$ and $\mu \in {\mathbb{H}}^{d}$ to $y{\in T}_{\mu }{\mathbb{H}}^{d}$, and (c) Project $y$ to $z\in {\mathbb{H}}^{d}$.

**Figure 3:**
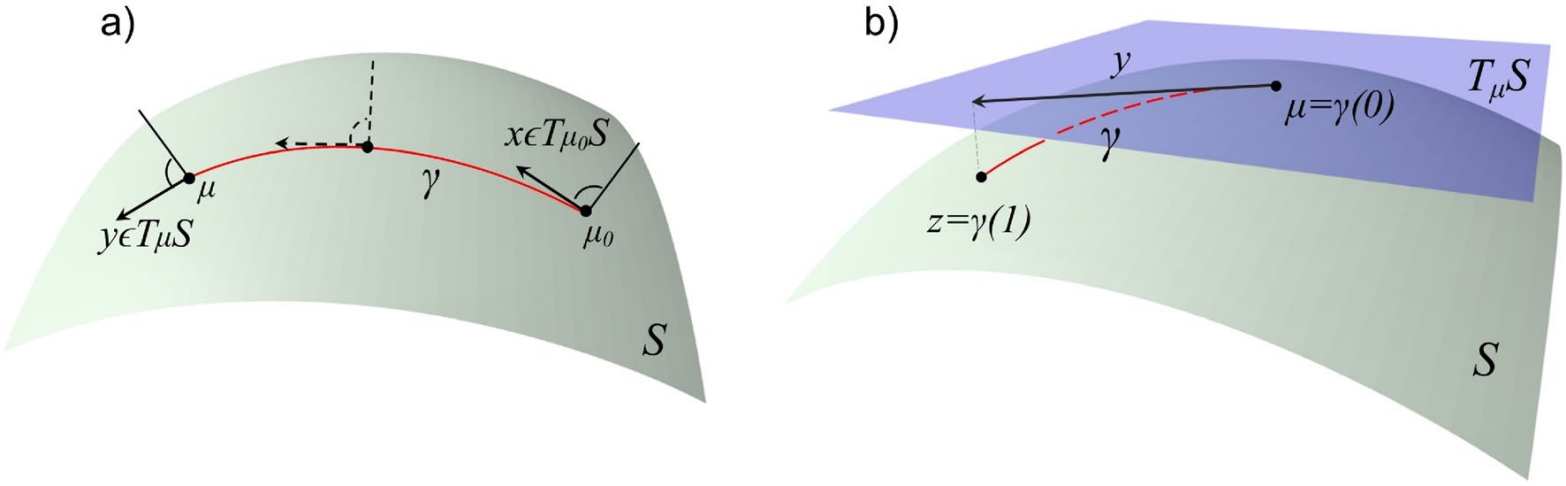
(**a**) Parallel transport of $x\in {T}_{{\mu }_{0}}S$ to $y\in {T}_{\mu }S$ along the geodesic $\gamma$, where ${\mu }_{0}=\gamma \left(0\right)$ and $\mu =\gamma \left(1\right)$. (**b**) The exponential map.

The step (b) is carried out using the parallel transport ${g}_{{\mu }_{0}\to \mu }:{T}_{{\mu }_{0}}{\mathbb{H}}^{d}\to {T}_{\mu }{\mathbb{H}}^{d}$ (Fig. 3a), defined by

11 $${g}_{{\mu }_{0}\to \mu }\left(x\right)=x+\frac{{\langle \mu +{\langle {\mu }_{0},\mu \rangle }_{\mathcal{L}}{\cdot \mu }_{0},x\rangle }_{\mathcal{L}}}{1-{\langle {\mu }_{0},\mu \rangle }_{\mathcal{L}}}\left({\mu }_{0}+\mu \right),$$

while the step (c) uses the exponential map $Ex{p}_{\mu }:{T}_{\mu }{\mathbb{H}}^{d}\to {\mathbb{H}}^{d}$ (Fig. 3b), defined by

12 $$Ex{p}_{\mu }\left(y\right)=\mathrm{cosh}\left({\Vert y\Vert }_{\mathcal{L}}\right)\mu +\mathrm{sinh}\left({\Vert y\Vert }_{\mathcal{L}}\right)\frac{y}{{\Vert y\Vert }_{\mathcal{L}}},$$

where $${\Vert y\Vert }_{\mathcal{L}}=\sqrt{{\langle y,y\rangle }_{\mathcal{L}}}$$.

It is not difficult to show that the length of the geodesic joining $\mu$ to $Ex{p}_{\mu }\left(y\right)$ on ${\mathbb{H}}^{d}$ is equal to ${\Vert y\Vert }_{\mathcal{L}}$, i.e., ${d}_{{\mathbb{H}}^{d}}\left(\mu ,Ex{p}_{\mu }\left(y\right)\right)={\Vert y\Vert }_{\mathcal{L}}$. The relationship between the probability densities $X\sim \mathcal{N}\left(0,\Sigma \right)$ and $Z\sim \mathcal{G}\left(\mu ,\Sigma \right)$ is

13 $$p\left(x\right)=p\left(z\right)\mathrm{det}\left({J}_{f}\right),$$

where $f=Ex{p}_{\mu }\circ {g}_{{\mu }_{0}\to \mu }$ and $\mathrm{det}\left({J}_{f}\right)$ denotes the determinant of the Jacobian ${J}_{f}=\left|\frac{\partial f}{\partial x}\right|$^35^. Finally, it can be shown that

14 $$\mathrm{ln}p\left(z\right)=\mathrm{ ln}p\left(x\right)-\left(d-1\right)\mathrm{ln}\frac{\mathrm{sinh}\left(r\right)}{r},$$

where $$r=\mathrm{arccosh}\left(-{\langle \mu ,z\rangle }_{\mathcal{L}}\right)$$^35^.

### The loss function

With the prior placed on $U$ and $V$, we return to calculating the posterior probability $p\left(U,V|R\right)$ through the Bayesian inference

15 $$p\left(U,V|R\right)\propto p\left(R|U,V\right)p\left(U|{\sigma }^{2}\right)p\left(V|{\sigma }^{2}\right).$$

Following the Euclidean matrix factorization, we take the logarithm of the posterior distribution (15) to arrive at the closed form expression for the loss function

16 $$\begin{aligned} L & =\sum_{i=1}^{m}\sum_{j=1}^{n}{w}_{i,j}\left[\mathrm{ln}\left(1+{e}^{-{d}_{\mathcal{L}}^{2}\left({u}^{i},{v}^{j}\right)}\right)+{r}_{i,j}{d}_{\mathcal{L}}^{2}\left({u}^{i},{v}^{j}\right)\right] \\ & \quad - \sum \limits_{i=1}^{m}\left[\mathrm{ln}p\left(\overline{{u}^{i}}\right)-\left(d-1\right)\mathrm{ln}\frac{\mathrm{sinh}\left({\Vert \overline{{u}^{i}}\Vert }_{\mathcal{L}}\right)}{{\Vert \overline{{u}^{i}}\Vert }_{\mathcal{L}}}\right] \\ & \quad -\sum \limits_{j=1}^{n}\left[\mathrm{ln}p\left(\overline{{v}^{j}}\right)-\left(d-1\right)\mathrm{ln}\frac{\mathrm{sinh}\left({\Vert \overline{{v}^{j}}\Vert }_{\mathcal{L}}\right)}{{\Vert \overline{{v}^{j}}\Vert }_{\mathcal{L}}}\right]. \end{aligned}$$

In the expression above, $p$ is the probability density function of the normal distribution $\mathcal{N}\left(0,{\upsigma }^{2}I\right)$ in the tangent space ${T}_{{\mu }_{0}}{\mathbb{H}}^{d}$ at the vertex ${\mu }_{0}=\left(0,\dots ,\mathrm{0,1}\right)$ and, for $x=\left({x}_{1},\dots ,{x}_{d},{x}_{d+1}\right)\in {\mathbb{H}}^{d}$,

17 $$\overline{x}={\mathit{Exp}}_{{\mu }_{0}}^{-1}x=\frac{\mathrm{arccosh}\left(-{\langle {\mu }_{0},x\rangle }_{\mathcal{L}}\right)}{\sqrt{{\langle {\mu }_{0},x\rangle }_{\mathcal{L}}^{2}-1}}\left(x+{\langle {\mu }_{0},x\rangle }_{\mathcal{L}}\cdot {\mu }_{0}\right)=\frac{\mathrm{arccosh}\left({x}_{d+1}\right)}{\sqrt{{x}_{d+1}^{2}-1}}\left({x}_{1},\dots ,{x}_{d},0\right).$$

Thus,

18 $$\mathrm{ln}p\left(\overline{x}\right)=-\frac{1}{{2\upsigma }^{2}}{\mathrm{arccosh}}^{2}{(x}_{d+1})+{C}_{1},$$

where ${C}_{1}$ is a constant. Moreover, since

19 $${\Vert \overline{x}\Vert }_{\mathcal{L}}=\mathrm{arccosh}\left(-{\langle {\mu }_{0},x\rangle }_{\mathcal{L}}\right)=\mathrm{arccosh}\left({x}_{d+1}\right),$$

It follows that

20 $$\frac{\mathrm{sinh}\left({\Vert \overline{x}\Vert }_{\mathcal{L}}\right)}{{\Vert \overline{x}\Vert }_{\mathcal{L}}}=\frac{\sqrt{{x}_{d+1}^{2}-1}}{\mathrm{arccosh}\left({x}_{d+1}\right)}.$$

Hence, our loss function has the following form:

21 $$\begin{aligned} L & =\sum_{i=1}^{m}\sum_{j=1}^{n}{w}_{i,j}\left[\mathrm{ln}\left(1+{e}^{-{d}_{\mathcal{L}}^{2}\left({u}^{i},{v}^{j}\right)}\right)+{r}_{i,j}{d}_{\mathcal{L}}^{2}\left({u}^{i},{v}^{j}\right)\right] \\& \quad +\sum \limits_{i=1}^{m}\left[{\alpha }_{U}{\mathrm{arccosh}}^{2}\left({u}_{d+1}^{i}\right)+\left(d-1\right)\mathrm{ln}\frac{\sqrt{{\left({u}_{d+1}^{i}\right)}^{2}-1}}{\mathrm{arccosh}\left({u}_{d+1}^{i}\right)}\right] \\ & \quad +\sum \limits_{j=1}^{n}\left[{\alpha }_{V}{\mathrm{arccosh}}^{2}\left({v}_{d+1}^{j}\right)+\left(d-1\right)\mathrm{ln}\frac{\sqrt{{\left({v}_{d+1}^{j}\right)}^{2}-1}}{\mathrm{arccosh}\left({v}_{d+1}^{j}\right)}\right]+C, \end{aligned}$$

where ${\alpha }_{U}=\frac{1}{{2\sigma }_{U}^{2}}$, ${\alpha }_{V}=\frac{1}{{2\sigma }_{V}^{2}}$ are trainable parameters and $C$ is a constant.

### Alternating gradient descent in hyperbolic space

Minimizing a real function defined in a $d$-dimensional Euclidean space ${\mathbb{R}}^{d}$ is routinely accomplished using the gradient descent technique. We adopt a similar method for finding the point $u\in {\mathbb{H}}^{d}$ of a local minimum of any real valued function $f:{\mathbb{H}}^{d}\to {\mathbb{R}}$^36,37^. For this strategy to work, the function $f$ must be defined is in the ambient space ${\mathbb{R}}^{d,1}$ of ${\mathbb{H}}^{d}$, as well as on ${\mathbb{H}}^{d}$. Specifically, given the initial value ${u=u}^{\left(0\right)}$ and a step size $\eta$, the gradient descent in hyperbolic space can be carried out by repeating the following steps:

1. Compute the gradient ${\nabla }_{u}^{{\mathbb{R}}^{d,1}}f$
2. Project ${\nabla }_{u}^{{\mathbb{R}}^{d,1}}f$ orthogonally to vector ${\nabla }_{u}^{{\mathbb{H}}^{d}}f\in {T}_{u}{\mathbb{H}}^{d}$
3. Set $${u}^{new}=Ex{p}_{u}\left(-\eta {\nabla }_{u}^{{\mathbb{H}}^{d}}f\right)$$

The gradient ${\nabla }_{u}^{{\mathbb{R}}^{d,1}}f$ in the ambient space ${\mathbb{R}}^{d,1}$ is a vector of partial derivatives

22 $${\nabla }_{u}^{{\mathbb{R}}^{d,1}}f=\left({\frac{\partial L}{\partial {x}_{1}}|}_{u} ,\dots ,{\frac{\partial L}{\partial {x}_{n}}|}_{u},-{\frac{\partial L}{\partial {x}_{d+1}}|}_{u}\right)$$

(note the negative sign of the last vector’s component).

The above representation of the gradient follows directly from its definition:

23 $$\forall v\in {\mathbb{R}}^{d,1}, {\langle {\nabla }_{u}^{{\mathbb{R}}^{d,1}}f,v\rangle }_{\mathcal{L}}={D}_{v}f\left(u\right).$$

The orthogonal projection from the ambient space onto the tangent space in (step 2 above) is given by

24 $${\nabla }_{u}^{{\mathbb{H}}^{d}}f={\nabla }_{u}^{{\mathbb{R}}^{d,1}}f+{\langle u,{\nabla }_{u}^{{\mathbb{R}}^{d,1}}f\rangle }_{\mathcal{L}}u.$$

We use the “alternating gradient descent” method to minimize the error function ${L}_{A,B}$ given in (21). The partial derivatives of ${L}_{A,B}$ are

25 $$\frac{\partial L}{\partial {u}_{k}^{i}}=2\sum_{j=1}^{n}{w}_{i,j}\left({p}_{i,j}-{r}_{i,j}\right){v}_{k}^{j}, k\le d$$

26 $$\begin{aligned} \frac{\partial L}{\partial {u}_{d+1}^{i}} & =2\sum_{j=1}^{n}{w}_{i,j}\left({r}_{i,j}-{p}_{i,j}\right){v}_{d+1}^{j} \\ & \quad + 2{\alpha }_{U}\frac{\mathrm{arccosh}\left({u}_{d+1}^{i}\right)}{\sqrt{{\left({u}_{d+1}^{i}\right)}^{2}-1}} \\ & \quad+ \left(d-1\right)\frac{{u}_{d+1}^{i}\mathrm{arccosh}\left({u}_{d+1}^{i}\right)-\sqrt{{\left({u}_{d+1}^{i}\right)}^{2}-1}}{\left[{\left({u}_{d+1}^{i}\right)}^{2}-1\right]\mathrm{arccosh}{(u}_{d+1}^{i})}\end{aligned}$$

**Figure 4:**
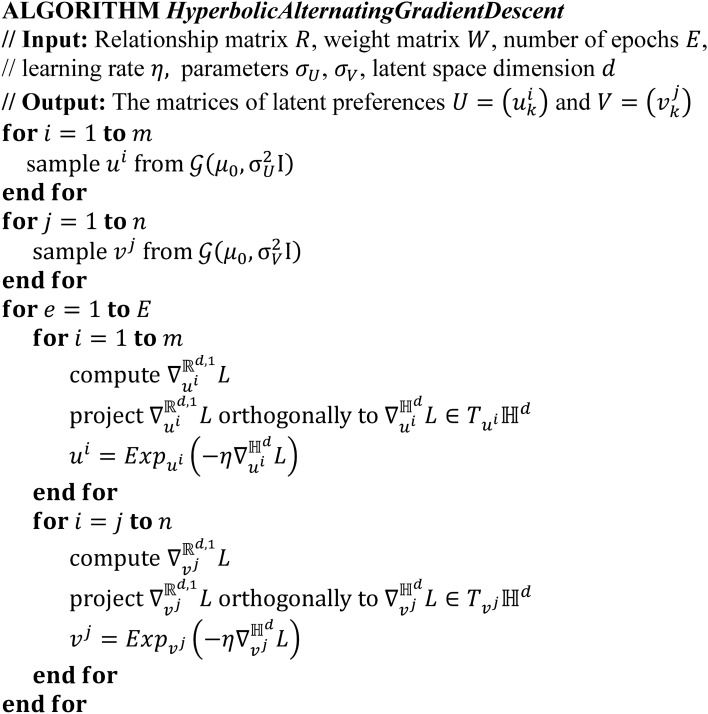
Pseudocode of the hyperbolic gradient descent procedure.

Figure 4 shows the pseudocode of our algorithm.

### Hyperbolic neighborhood regularization and cold-start

A standard way to increase the accuracy of relationship inference between the elements of two biological domains $A$ and $B$ is to employ the so-called *neighborhood regularization*. The goal is to ensure that similar entities from $A$ are in relationship with similar entities from $B$ (e.g., similar drugs interact with similar genes). To achieve this, we extend the Euclidean neighborhood regularization method^21,38^ to ${\mathbb{H}}^{d}$ by adding the following term to the loss function $L$(21):

27 $${\beta }_{U}\sum_{i=1}^{m}\sum_{j=1}^{m}{s}_{i,j}{d}_{\mathcal{L}}^{2}\left({u}^{i},{u}^{j}\right)+{\beta }_{V}\sum_{i=1}^{n}\sum_{j=1}^{n}{t}_{i,j}{d}_{\mathcal{L}}^{2}\left({v}^{i},{v}^{j}\right),$$

where ${s}_{i,j}$ (respectively ${t}_{i,j}$) is the value reflecting the similarity between ${a}^{i}$ and ${a}^{j}$ (respectively ${b}^{i}$ and ${b}^{j}$) and ${\beta }_{U}$, ${\beta }_{V}$ are trainable (neighborhood regularization) parameters.

A separate procedure is needed to address the “cold-start” problem i.e., the arrival of a new node (a node with no known relationships to other nodes). In the setting of drug-target interaction prediction, this procedure is used to predict targets for new compounds (such as a chemical in pre-clinical studies) and vice versa.

For the hyperbolic cold-start, we use a hyperbolic variant of the Euclidean weighted-profile method^21,31,33^. Specifically, the latent vector ${u}^{i}\in {\mathbb{R}}^{d}$ for a drug ${a}^{i}\in A$ that does not interact with any protein ${b}^{j}\in B$ (i.e., the ${i}^{th}$ row of $R$ is empty) is computed as the weighted combination of the rows ${u}^{j}\in U$ most similar to ${u}^{i}$. Specifically,

28 $${u}^{i}=\frac{1}{SM}\sum_{j=1}^{J}{s}_{i,j}{u}^{j},$$

where $SM=\sum_{j=1}^{J}{s}_{i,j}$ and $J$ is a pre-defined number of nearest neighbors. The hyperbolic center of mass ${u}^{i}$ is computed as in Law et al.^39^.

## Results

### Benchmarking experiments

We benchmarked the hyperbolic matrix factorization on four drug-target interaction test sets, specifically Nr, Gpcr, Ion, and Enz^40^, using four traditional classification measures, namely the area under the receiver operating characteristics curve (AUC), the area under the precision-recall curve (AUPR), precision at top ten (PREC@10), and the average precision (AP). An extensive grid search is employed to train the parameters of each method (see the Supplementary Data).

In our first benchmark, we assessed the advantage of the basic logistic hyperbolic matrix factorization over the classical Euclidean matrix factorization (as implemented in the popular NRLMF method^21^), in absence of any side-information (i.e., the pairwise drug and the pairwise protein similarity). As described in the “Methods” section, the hyperbolic method is conceptually the same as the Euclidean method, but it uses ${-d}_{\mathcal{L}}^{2}\left(x,y\right)=2+2{\langle x,y\rangle }_{\mathcal{L}}$ in place of $\langle x,y\rangle$ and uses the pseudo-hyperbolic Gaussian distribution (10) in place of the Gaussian prior.

We submit each method (Euclidean and hyperbolic) to ten rounds of the fivefold cross-validation (CV) test (also known as CVP test^22^). In each CV round, the data set under consideration (i.e., the drug-target association matrix) is randomly split into 5 groups. Each group is used once as test data, while the remaining four groups represent training data. Hence, every (interacting and non-interacting) drug-target pair is scored once in each CV round. The final classification score (AUC, AUPR, PREC@10, AP) assigned to each DTI prediction method is computed by averaging classification scores obtained across different CV rounds.

As seen in Table 1, the bare-bone hyperbolic matrix factorization routinely outperforms the bare-bone Euclidean factorization in identifying four types of drug targets (Nr, Gpcr, Ion, and Enz) and across fundamentally different classification measures (AUC, AUPR, PREC10, AP).

**Table 1:** Comparison of the basic (no side-information or profile-weighting) Euclidean and hyperbolic logistic matrix factorization algorithm (as implemented in the NRLMF method).

	AUC	AUPR	PREC10	AP
Nr				
Euc	0.771 ± 0.009	0.340 ± 0.015	0.551 ± 0.010	0.461 ± 0.020
Hyp	**0.850** ± 0.007	**0.433** ± 0.023	**0.647** ± 0.020	**0.567** ± 0.028
Gpcr				
Euc	0.884 ± 0.006	0.571 ± 0.011	0.983 ± 0.021	0.987 ± 0.024
Hyp	**0.904** ± 0.002	**0.612** ± 0.007	**1.000** ± 0.000	**1.000** ± 0.000
Ion				
Euc	0.968 ± 0.002	0.842 ± 0.005	1.000 ± 0.000	1.000 ± 0.000
Hyp	**0.975** ± 0.003	**0.861** ± 0.001	1.000 ± 0.000	1.000 ± 0.000
Enz				
Euc	0.951 ± 0.001	0.770 ± 0.002	1.000 ± 0.000	1.000 ± 0.000
Hyp	**0.966** ± 0.001	**0.809** ± 0.003	1.000 ± 0.000	1.000 ± 0.000

The results are obtained using ten rounds of fivefold cross-validation. Better results are shown in bold.

**Figure 5:**
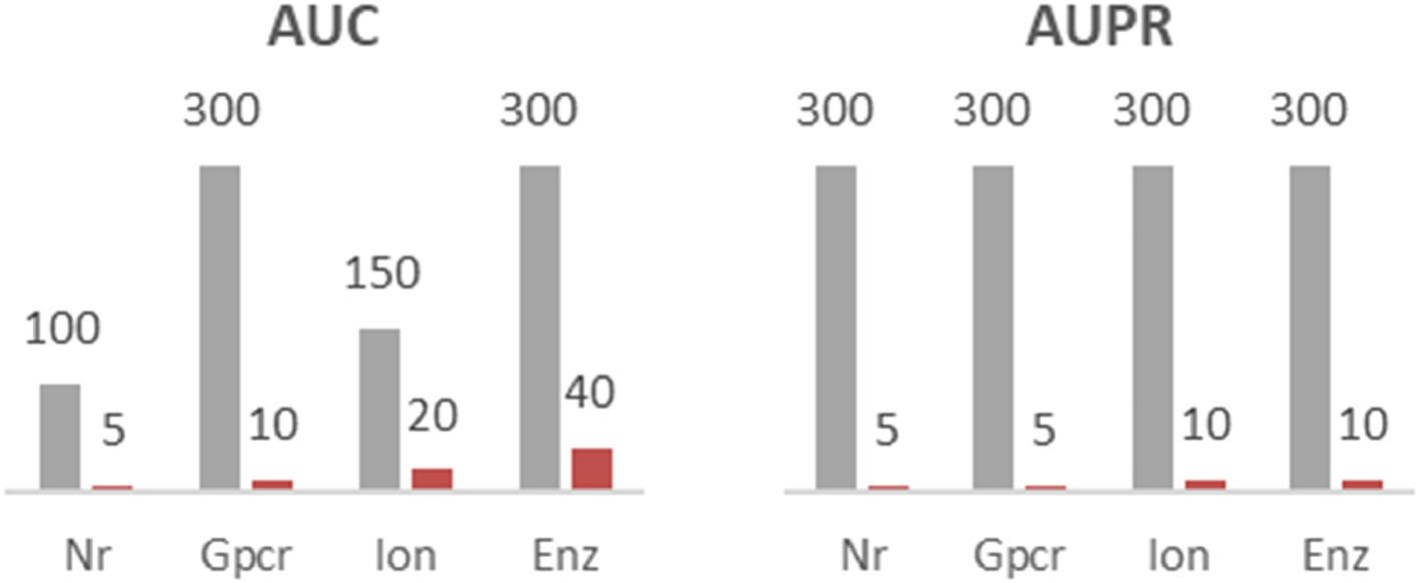
Optimal Euclidean rank (gray) and the hyperbolic rank (red) yielding the same or better AUC and the AUPR scores. Similar results were obtained using the PREC10 metric and the AP metric.

Interestingly, the hyperbolic matrix factorization achieves superior accuracy at latent dimensions that are by an order of magnitude smaller compared to dimensions needed for an optimal Euclidean embedding. Specifically, optimal Euclidean factorization is most often achieved at ranks exceeding 150. In contrast, most of the time, hyperbolic factorization needs only 5 or 10 latent features to achieve the same or better classification scores (Fig. 5). We view this as additional evidence that the hyperbolic space is the native space of biological networks.

In our second test, we allow both methodologies to use drug and protein homophily information to boost the prediction accuracy. In the classical (Euclidean) setting, we incorporate side-information precisely as done in the NRLMF method^21^. The hyperbolic algorithm uses the same general formula (27), but employs the hyperbolic distances in place of the Euclidean distances. As seen in Table 2, the Euclidean factorization erases some head-start advantage of hyperbolic factorization in the fivefold CVP test, albeit at much higher latent dimensions. This is somewhat expected, as the side information enables the Euclidean method to approach the theoretical limits on the accuracy that can be achieved on the four noisy, sparse, and biased test sets used in our study.

**Table 2:** Accuracy of the full-blown Euclidean and hyperbolic logistic matrix factorizations in predicting drug-target interactions (10 rounds of fivefold CV test).

	AUC	AUPR	PREC10	AP
Nr				
Euc	0.954 ± 0.002	0.642 ± 0.013	0.808 ± 0.044	0.755 ± 0.006
Hyp	**0.970** ± 0.004	**0.697** ± 0.019	**0.874** ± 0.010	**0.848** ± 0.028
Gpcr				
Euc	0.962 ± 0.002	0.677 ± 0.010	0.913 ± 0.056	0.872 ± 0.078
Hyp	**0.976** ± 0.002	**0.710** ± 0.017	**0.989** ± 0.010	**0.984** ± 0.014
Ion				
Euc	0.984 ± 0.002	0.868 ± 0.005	0.948 ± 0.017	0.890 ± 0.018
Hyp	**0.991** ± 0.001	**0.890** ± 0.010	**1.000** ± 0.000	**1.000** ± 0.000
Enz				
Euc	0.983 ± 0.001	0.879 ± 0.006	1.000 ± 0.000	1.000 ± 0.000
Hyp	**0.991** ± 0.001	**0.899** ± 0.001	1.000 ± 0.000	1.000 ± 0.000

Both methods are allowed to take advantage of the side-information and profile weighting.

Significantly better values are shown in bold.

For a more thorough analysis, we also carried out the above benchmarks using tenfold cross validation. The results of our tenfold CV tests are shown in the Supplementary Tables 1 and 2. Depending on a test set under consideration, tenfold cross validation might be a more meaningful experiment as removing only 10% of the existing network links (as opposed to 20% in a fivefold CV) preserves important structural features of the target network^41,42^.

While the first two benchmarks help gain insight into the value added by different components of the loss-function, our final benchmark compares the two techniques in the most important and the most difficult cold-start setting. In this experiment, known as Leave-One-Out Cross-Validation (LOOCV), we hide (zero out) and then try to recover all interactions of every drug under consideration. Specifically, for each drug *d*, we hide (zero out) and then try to recover all interactions (known and unknown) of *d* with all proteins in the data set. Thus, LOOCV can be viewed as a (non-stochastic) variant of a (single round) *m*-cross validation procedure, where *m* is the number of drugs.

To better assess the performance of hyperbolic embedding, we include in the LOOCV benchmark two additional state-of-the-arts methods, namely, DNILMF^22^, and NGN^24^. The DNILMF method is like NRLMF, but it incorporates drug and protein homophily directly into the formula for ${p}_{i,j}$ (6). Moreover, it employs a nonlinear diffusion technique to construct pairwise drug and protein similarity matrices^22^. The NGN method is also similar in spirit to NRLMF, but it builds a neighborhood-based global network model instead of learning drug and target features separately^24^.

We constructed a hyperbolic variant of each technique by simply replacing the Euclidean dot product with the negative Lorentzian distance and by replacing the Gaussian prior by the wrapped normal distribution in the hyperbolic space (as discussed in the “Methods” section).

As seen in Table 3, the hyperbolic matrix factorization improves the accuracy of current techniques in predicting protein targets for new compounds, such as the chemicals in preclinical studies or clinical trials. In addition, the Supplementary Table 3 shows that our method improves DTI predictions on isolated samples, namely drug-target pairs $(d,t)$, where $d$ does not have interacting targets (other than $t$) and $t$ does not have interacting drugs (other than $d$).

**Table 3:** The accuracy of the Euclidean vs. hyperbolic variants of different matrix factorization methods in the Leave-One-Out Cross-Validation (LOOCV) benchmark.

	AUC	AUPR	PREC10	AP
NRLMF				
Euc	0.905	0.243	0.495	0.549
Hyp	**0.926**	**0.258**	**0.557**	**0.612**
DNILMF				
Euc	0.895	0.239	0.489	0.546
Hyp	**0.923**	**0.262**	**0.562**	**0.622**
NGN				
Euc	0.895	0.243	0.506	0.554
Hyp	**0.925**	**0.263**	**0.558**	**0.614**

Significantly better values are shown in bold.

### Additional tests

Recent years have seen the developments of machine learning algorithms for different biological relationship inference tasks^43–46^. While many of those methods can be tailored to provide predictions of drug-target interactions, it would be unrealistic to benchmark them all against the methodology presented in this article. Supplementary Table 4 provides the comparison of our technique against the SVM-based algorithm BLM^47^ and the GRGMF—a matrix factorization algorithm^48^.

We were also interested in how our method fares against the Cannistraci’s methods^49^ based on the local-community-paradigm (LCP). These methods are simple to interpret as they use a combination of node similarity metrics (directly observable in a bipartite drug-target network), such as the number of common neighbors (CN) and the number of links between those neighbors (LCL). Aside from exhibiting the accuracy superior to that of other unsupervised drug-target link prediction algorithms (and comparable to accuracies of supervised algorithms), Cannistraci’s methods are extremely fast (Supplementary Fig. 1) and thus ideally suited for the task of link prediction in large networks. The results of our comparison with the LCP-based methods are shown in the Supplementary Tables 5 and 6.

While our project was, in part, inspired by the recent studies on hyperbolic network embedding, most of those methods, such as Coalescent Embedding (CE)^14^, were not specifically tailored for the DTI prediction task. To make a meaningful comparison with CE, we had to first place the two algorithms on the same ground. More precisely, in our tests the inference by CE was conducted based upon the hyperbolic distances between drugs and targets (closer objects are more likely to interact) computed from the coalescent embedding of the drug-target interaction network in the Poincaré disk. We also restricted the embedding dimension in our method to 2 since CE preferably uses the Poincaré disk as the latent space. The classification scores achieved by the two techniques are presented in the Supplementary Tables 7 and 8. We emphasize that, due to the methods’ modifications mentioned above, the benchmarking results shown in the supplementary material should be interpreted with caution.

In a quest for high accuracy, some algorithms for DTI prediction utilize biomedical knowledge beyond the protein amino-acid sequences and drug chemical structures, including the information on adverse drug reactions, drug-disease and protein-disease associations, drug-induced gene expression profiles, protein–protein interactions, etc. Such a rich input often leads to information leak, presenting a challenge in evaluating these methods in a classical drug discovery setting where (typically) only a chemical structure of the drug and the primary sequence of the gene is known upfront.

Recent years have also seen the development of methods for drug-target affinity (DTA) prediction^50–53^. In contrast to DTI prediction methods, DTA algorithms utilize drug-target binding affinity scores and treat DTI as a regression (rather than a binary classification) problem. Moreover, unlike DTI methods, DTA algorithms are typically evaluated on Davis^54^ and KIBA^55^ datasets using Concordance Index (CI), Mean Squared Error (MSE), and similar metrics for regression classification tasks. In fact, aside from KronRLS^56^, very few DTA methods have been assessed in standard DTI benchmarks. While the direct comparison with DTA methods is beyond the scope of this paper, a quick look at the AUPR values in a cross-validation test published by KronRLS authors (Nr: 0.528, Gpcr: 0.602, Ion: 0.765, Enz: 0.829) and the corresponding values computed in our benchmark (Nr: 0.697, Gpcr: 0.710, Ion: 0.890, Enz: 0.899) provide some insight (albeit indirect) into potential benefits of utilizing hyperbolic space to predict drug-target binding affinities.

## Discussion and conclusion

Matrix factorization is one of the main techniques used in computational systems biology to uncover relationships between the elements from a pair of biological domains. The technique works by representing the biological objects as points in a low dimensional (latent) space in a way that best explains the input set of known interactions. More precisely, the input matrix of know associations is completed by approximating it as a product of two lower dimensional matrices.

Past research in computational systems biology, including matrix factorization techniques, has taken the Euclidean geometry of the biological space for granted. This has been convenient due to the availability of advanced analytic, numerical, statistical and machine learning procedures in the Euclidean space. However, recent theoretical studies suggest that the hyperbolic geometry, rather than Euclidean, underpins all complex networks in general and the biological networks in particular. Therefore, a radical shift in data representation is necessary to obtain an undistorted view of the biological space and, in turn, ensure further progress in systems biology and related fields.

We have developed and benchmarked a technique for a probabilistic hyperbolic matrix factorization and applied it to predict drug-target interactions. We demonstrate that the Lorentzian model of hyperbolic space allows for a closed form expression of the key transformations and techniques required for latent space dimensionality reduction. Our method builds upon recent advances in the development of probabilistic models and numerical optimization in hyperbolic space to learn an optimal embedding and to compute the probabilities of drug-target interactions. Our benchmarking tests demonstrate a significant increase in accuracy and a drastic reduction in latent space dimensionality of hyperbolic embedding compared to Euclidean embedding. These findings reaffirm the negative curvature of the native biological space.

Although a (bipartite drug-target) hyperbolic network embedding arises as a byproduct of hyperbolic matrix factorization, our focus is on prioritizing targets for a given drug (and vice versa). To better assisting structure-based drug discovery, DTI prediction methods focus more on identifying a handful of targets with strong binding affinities and much less on prioritizing many remaining targets with weak interactions (this also explains why the AUPR-like metrics are preferred in computational systems biology). To achieve this goal, DTI prediction methods are willing to distort the network structure away from the immediate neighbors of each drug in order to better model the network in the vicinities of drugs. In our methods, the distortion occurs each time a weighted profile is constructed to address the cold-start problem.

There are several aspects of hyperbolic matrix factorization that this study has not explored in detail, including optimal procedure for gradient descent in hyperbolic space. In contrast to decades of research on Euclidean numerical analysis techniques, the methods for numerical optimization in the hyperbolic space are few and far between. The main difficulty is the numerical instability of the hyperbolic gradient descent in vicinity of cliffs^57^. In this study, we applied a simple heuristic intervention to combat the explosion in the magnitude of the hyperbolic gradient. For our optimization method to converge to a local minimum, we carried out three iterations of the gradient descent procedure, lowering the learning rate on the fly and clipping the gradient if necessary. We believe that further research in this area will add significant value to hyperbolic embedding and inference methods.

Our model uses the same hyperbolic space to represent both drugs and proteins. This widely used approach^58–60^ is applied in our study due to simplicity of algorithm design and the fact that heterogeneous networks are shown to have a metric structure with an effective hyperbolic geometry underneath^61^. However, alternative approaches are also worthwhile considering. Viewing biomedical entities (in our case drugs and proteins) as objects residing in spaces of different dimension and curvature, the bipartite graph of their relationships can be realized in the hyperbolic product space^62^. Finding the proper dimension and the curvature of the space that underlines each biological domain is expected to result in a more accurate latent representation and, in turn, more accurate relationship prediction.

## Supplementary Information

Supplementary Information.

## Data Availability

The code and test sets are available in the github repository https://github.com/poleksic/Hyperbolic_MF.
