# Supplementary material for: Hyperbolic matrix factorization improves prediction of drug-target associations
Source: Sci Rep. 2023 Jan 18;13:959. doi: 10.1038/s41598-023-27995-5 (PMC9849222; doi:10.1038/s41598-023-27995-5)
Supplement: Supplementary file 1 — Supplementary Information. [file 41598_2023_27995_MOESM1_ESM.docx]

Hyperbolic matrix factorization improves prediction of drug-target associations

**Supplementary material**

**Algorithm parameters**

Each method submitted to our benchmarks explores a fine grid of parameters to optimize the classification scores (AUC, AUPR, PREC@10, AP). For best performance, we optimized the Euclidean methods (NRLMF, DNILMF, and NGN) on the parameter grids chosen by their authors. These parameter sets can be found in the respective publications [^[[1]](#endnote-1)^,^[[2]](#endnote-2)^,^[[3]](#endnote-3)^].

The hyperbolic algorithms (hyperbolic NRLMF, hyperbolic DNILMF, and hyperbolic NGN) employ the same types of parameters as their Euclidean counterparts. However, for optimal performance, these parameters are selected differently. For instance, an optimal rank parameter for the Euclidean methods is often $\geq100$, which is an order of magnitude larger than an optimal hyperbolic rank.

Below, we specify the parameter space explored by the hyperbolic matrix factorization techniques.

- Rank $d$ is chosen from the set {5, 10, 20, 40}
- Learning rate is set to $\eta=2^{-2}$
- The neighborhood regularization parameters $\beta_{U}$ and $\beta_{V}$ are chosen from the set {$2^{-4},2^{-3},2^{-2},2^{-1},2^{0},2^{1}\}$
- The number of neighbors $J$ for inferring new drug/target profile is chosen from {$2^{0},2^{1},2^{2},2^{3}\}$
- The parameter used to control the importance levels of observed interactions c is chosen from the set {$2^{0},2^{2},2^{4}\}$
- The regularization parameters are set to $\alpha_{U}=\alpha_{V}=2^{-1}$

In addition, we introduce the parameter $\theta$ to simulate matrix factorization in the hyperbolic space $H^{d,\theta}$ of curvature ${-1}/\theta$, $\theta>0$, defined as

$H^{d,\theta}=\{x\in R^{d,1}\vee\left\langle x,x \right\rangle_{L}=-\theta,x_{d+1}>0\}$.

For a given curvature $\theta$, the logistic probability in (given by the formula 6 of the Methods section) generalizes to

$p_{i,j}=p\left( r_{i,j}=1 | u^{i},v^{j} \right)=\frac{exp\left( -d_{L,\theta}^{2}\left( u^{i},v^{j} \right) \right)}{1+exp\left( -d_{L,\theta}^{2}\left( u^{i},v^{j} \right) \right)}$,

where $d_{L,\theta}^{2}\left( x,y \right)$ is the generalized Lorentzian distance [^[[4]](#endnote-4)^] between the points $x,y\in H^{d,\theta}$, defined as

$d_{L,\theta}^{2}\left( x,y \right)=\left\langle x-y,x-y \right\rangle_{L}=-2\theta-2\left\langle x,y \right\rangle_{L}$.

Our current implementation uses a slightly simpler formula, namely

$p_{i,j}=\frac{exp\left( \left\langle u^{i},v^{j} \right\rangle+\theta\right)}{1+exp\left( \left\langle u^{i},v^{j} \right\rangle+\theta\right)}$,

optimizing the parameter $\theta$ over the set $\left\{ 2,3,5,8,12 \right\}.$

Supplementary Table 1. Comparison of the basic (no side-information or profile-weighting) Euclidean and hyperbolic logistic matrix factorization algorithm (as implemented, for instance, in the NRLMF method). The results are obtained using 10 rounds of 10-fold cross-validation.

|  | AUC | AUPR | PREC10 | AP |
| --- | --- | --- | --- | --- |
| Nr | | | | |
| *Euc* | 0.786±0.008 | 0.346±0.021 | 0.467±0.029 | 0.414±0.026 |
| *Hyp* | **0.856**±0.016 | **0.440**±0.021 | **0.526**±0.013 | **0.478**±0.025 |
| Gpcr | | | | |
| *Euc* | 0.896±0.003 | 0.611±0.018 | 0.984±0.010 | 0.988±0.021 |
| *Hyp* | **0.921**±0.002 | **0.659**±0.006 | **1.000**±0.000 | **1.000**±0.000 |
| Ion | | | | |
| *Euc* | 0.976±0.002 | 0.871±0.004 | 1.000±0.000 | 1.000±0.000 |
| *Hyp* | **0.979**±0.000 | **0.880**±0.003 | 1.000±0.000 | 1.000±0.000 |
| Enz | | | | |
| *Euc* | 0.953±0.001 | 0.787±0.002 | 1.000±0.000 | 1.000±0.000 |
| *Hyp* | **0.974**±0.002 | **0.838**±0.021 | 1.000±0.000 | 1.000±0.000 |

Supplementary Table 2. Accuracy of the full-blown Euclidean and hyperbolic logistic matrix factorizations in predicting drug-target interactions in 10 rounds of the 10-fold CV test. Both methods are allowed to take advantage of the side-information and profile weighting.

|  | AUC | AUPR | PREC10 | AP |
| --- | --- | --- | --- | --- |
| Nr | | | | |
| *Euc* | 0.957±0.003 | 0.656±0.036 | 0.752±0.005 | 0.723±0.018 |
| *Hyp* | **0.976**±0.003 | **0.699**±0.015 | **0.771**±0.020 | **0.757**±0.016 |
| Gpcr | | | | |
| *Euc* | 0.970±0.001 | 0.760±0.009 | 0.950±0.010 | 0.913±0.021 |
| *Hyp* | **0.980**±0.001 | **0.769**±0.009 | **0.980**±0.020 | **0.969**±0.013 |
| Ion | | | | |
| *Euc* | 0.988±0.001 | 0.910±0.010 | 0.973±0.015 | 0.947±0.031 |
| *Hyp* | **0.992**±0.000 | **0.918**±0.003 | **1.000**±0.000 | **1.000**±0.000 |
| Enz | | | | |
| *Euc* | 0.984±0.001 | 0.887±0.002 | 1.000±0.000 | 1.000±0.000 |
| *Hyp* | **0.995**±0.002 | **0.905**±0.004 | 1.000±0.000 | 1.000±0.000 |

Supplementary Table 3. Comparison of the HNRLMF (*Hyp*) method and the NRLMF (*Euc*) method on “isolated samples” i.e., drug target pairs $\boldsymbol{(d,t)}$, where $\boldsymbol{d}$ has no other interacting targets $\boldsymbol{t}$ has no other interacting drugs. To compute classification scores, non-interacting drug-target pairs are selected at random so that the ratio of interacting and non-interacting pairs matches their overall ratio in the data set under consideration.

|  | Nr | Gpcr | Ion | Enz |
| --- | --- | --- | --- | --- |
| AUC | | | | |
| *NRLMF* | 0.884 | 0.800 | 0.786 | 0.8410 |
| *HNRLMF* | **1.000** | **0.983** | **1.000** | **0.997** |
| AUPR | | | | |
| *NRLMF* | 0.209 | 0.069 | 0.071 | 0.039 |
| *HNRLMF* | **0.667** | **0.557** | **0.250** | **0.646** |

Supplementary Table 4. Comparison of the accuracy of HNRLMF (*Hyp*) and the published accuracy of BLM and GRGMF methods in a 10-fold cross validation test.

|  | Nr | Gpcr | Ion | Enz |
| --- | --- | --- | --- | --- |
| AUC | | | | |
| *BLM* | 0.841 | 0.944 | 0.972 | 0.968 |
| *GRGMF* | 0.917 | 0.958 | 0.988 | 0.982 |
| *HNRLMF* | **0.976** | **0.980** | **0.992** | **0.995** |
| AUPR | | | | |
| *BLM* | 0.584 | 0.650 | 0.832 | 0.852 |
| *GRGMF* | - | - | - | - |
| *HNRLMF* | **0.699** | **0.769** | **0.918** | **0.905** |

Supplementary Table 5. Comparison of the bare-bone (no pairwise drug or protein similarity information) hyperbolic matrix factorization method with the LCP-based methods in 10 rounds of 5-fold cross validation experiment. The results of LCP methods were obtained by running the LCP program published by its authors [^[[5]](#endnote-5)^].

|  | AUC | AUPR | PREC10 | AP |
| --- | --- | --- | --- | --- |
| Nr | | | | |
| *CAR* | 0.670±0.017 | 0.299±0.029 | 0.520±0.042 | 0.415±0.055 |
| *CJC* | 0.674±0.021 | 0.307±0.031 | 0.420±0.072 | 0.247±0.049 |
| *CPA* | 0.504±0.035 | 0.244±0.036 | 0.522±0.039 | 0.403±0.065 |
| *CAA* | 0.667±0.016 | 0.310±0.036 | 0.537±0.084 | 0.426±0.081 |
| *CRA* | 0.670±0.018 | 0.344±0.029 | 0.556±0.037 | 0.469±0.041 |
| *Hyp* | **0.850**±0.007 | **0.433**±0.023 | **0.647**±0.020 | **0.567**±0.028 |
| Gpcr | | | | |
| *CAR* | 0.820±0.004 | 0.495±0.010 | 0.980±0.013 | 0.973±0.019 |
| *CJC* | 0.824±0.005 | 0.515±0.010 | 0.094±0.019 | 0.047±0.014 |
| *CPA* | 0.745±0.008 | 0.475±0.008 | 0.966±0.016 | 0.945±0.029 |
| *CAA* | 0.819±0.004 | 0.515±0.011 | 0.982±0.011 | 0.979±0.013 |
| *CRA* | 0.825±0.006 | 0.548±0.013 | 0.990±0.014 | 0.986±0.020 |
| *Hyp* | **0.904**±0.002 | **0.612**±0.007 | **1.000**±0.000 | **1.000**±0.000 |
| Ion | | | | |
| *CAR* | 0.905±0.002 | 0.673±0.005 | 0.994±0.010 | 0.992±0.013 |
| *CJC* | 0.908±0.002 | 0.716±0.005 | 0.040±0.062 | 0.027±0.062 |
| *CPA* | 0.882±0.006 | 0.674±0.012 | 0.996±0.008 | 0.995±0.010 |
| *CAA* | 0.914±0.003 | 0.732±0.006 | 0.998±0.006 | 0.998±0.007 |
| *CRA* | 0.916±0.003 | 0.730±0.006 | 0.934±0.027 | 0.895±0.045 |
| *Hyp* | **0.975**±0.003 | **0.861**±0.001 | **1.000**±0.000 | **1.000**±0.000 |
| Enz | | | | |
| *CAR* | 0.882±0.002 | 0.671±0.003 | 0.978±0.011 | 0.953±0.028 |
| *CJC* | 0.885±0.003 | 0.671±0.003 | 0.038±0.026 | 0.007±0.005 |
| *CPA* | 0.812±0.005 | 0.649±0.005 | 0.980±0.016 | 0.965±0.031 |
| *CAA* | 0.884±0.001 | 0.710±0.003 | 0.982±0.011 | 0.973±0.017 |
| *CRA* | 0.883±0.003 | 0.738±0.005 | 0.992±0.010 | 0.981±0.026 |
| *Hyp* | **0.966**±0.001 | **0.809**±0.003 | **1.000**±0.000 | **1.000**±0.000 |

Supplementary Table 6. Comparison of the bare-bone (no pairwise drug or protein similarity information) hyperbolic matrix factorization method with the LCP-based methods in 10 rounds of 10-fold cross validation experiment.

|  | AUC | AUPR | PREC10 | AP |
| --- | --- | --- | --- | --- |
| Nr | | | | |
| *CAR* | 0.673±0.025 | 0.250±0.036 | 0.400±0.036 | 0.329±0.040 |
| *CJC* | 0.689±0.017 | 0.269±0.020 | 0.406±0.029 | 0.332±0.029 |
| *CPA* | 0.491±0.027 | 0.241±0.024 | 0.414±0.033 | 0.341±0.029 |
| *CAA* | 0.677±0.016 | 0.297±0.028 | 0.423±0.024 | 0.355±0.028 |
| *CRA* | 0.679±0.018 | 0.288±0.024 | 0.426±0.033 | 0.359±0.029 |
| *Hyp* | **0.856**±0.016 | **0.440**±0.021 | **0.526**±0.013 | **0.478**±0.025 |
| Gpcr | | | | |
| *CAR* | 0.825±0.002 | 0.500±0.007 | 0.935±0.020 | 0.921±0.024 |
| *CJC* | 0.833±0.005 | 0.529±0.007 | 0.590±0.031 | 0.397±0.040 |
| *CPA* | 0.752±0.006 | 0.495±0.007 | 0.933±0.029 | 0.921±0.033 |
| *CAA* | 0.833±0.003 | 0.539±0.005 | 0.961±0.011 | 0.955±0.011 |
| *CRA* | 0.834±0.002 | 0.562±0.006 | 0.961±0.022 | 0.953±0.026 |
| *Hyp* | **0.921**±0.002 | **0.659**±0.006 | **0.997**±0.006 | **0.997**±0.006 |
| Ion | | | | |
| *CAR* | 0.911±0.003 | 0.683±0.004 | 0.983±0.007 | 0.975±0.010 |
| *CJC* | 0.912±0.003 | 0.725±0.004 | 0.796±0.042 | 0.603±0.055 |
| *CPA* | 0.890±0.003 | 0.679±0.003 | 0.985±0.008 | 0.981±0.012 |
| *CAA* | 0.920±0.003 | 0.744±0.004 | 1.000±0.000 | 1.000±0.000 |
| *CRA* | 0.924±0.004 | 0.738±0.006 | 0.933±0.018 | 0.889±0.019 |
| *Hyp* | **0.979**±0.000 | **0.880**±0.003 | 1.000±0.000 | 1.000±0.000 |
| Enz | | | | |
| *CAR* | 0.891±0.002 | 0.676±0.002 | 0.973±0.011 | 0.950±0.023 |
| *CJC* | 0.893±0.002 | 0.676±0.003 | 0.049±0.015 | 0.022±0.008 |
| *CPA* | 0.820±0.005 | 0.662±0.003 | 0.978±0.011 | 0.964±0.020 |
| *CAA* | 0.892±0.002 | 0.719±0.002 | 0.981±0.017 | 0.968±0.025 |
| *CRA* | 0.894±0.002 | 0.753±0.003 | 0.988±0.008 | 0.978±0.014 |
| *Hyp* | **0.974**±0.002 | **0.838**±0.021 | **1.000**±0.000 | **1.000**±0.000 |

Supplementary Table 7. Performance of the bare-bone hyperbolic matrix factorization (*Hyp* 2D) carried out in the 2-dimensional hyperbolic space and different variants of the 2D Coalescent Embedding (CE) algorithm in 10 rounds of 5-fold CV benchmark. All methods were given the same adjacency matrices of drug-target interactions graphs as input. The CE methods use the Laplacian Eigenmaps (LE) dimension reduction with different combinations of network pre-weighting, namely original (ORG), reverse (REV), RA1, and RA2. These methods can be configured to either preserve the angular distances (OD) or apply the equidistant angular adjustment (EA).

|  | AUC | AUPR | PREC10 | AP |
| --- | --- | --- | --- | --- |
| Nr | | | | |
| ORG LE OD | 0.521±0.014 | 0.161±0.018 | 0.360±0.039 | 0.229±0.041 |
| ORG LE EA | 0.710±0.012 | 0.193±0.018 | 0.406±0.038 | 0.300±0.038 |
| REV LE OD | 0.512±0.011 | 0.162±0.019 | 0.356±0.040 | 0.226±0.041 |
| REV LE EA | 0.718±0.010 | 0.195±0.025 | 0.402±0.059 | 0.303±0.061 |
| RA1 LE OD | 0.489±0.009 | 0.123±0.009 | 0.255±0.029 | 0.105±0.021 |
| RA1 LE EA | 0.707±0.018 | 0.161±0.011 | 0.317±0.036 | 0.158±0.041 |
| RA2 LE OD | 0.517±0.014 | 0.127±0.015 | 0.242±0.052 | 0.106±0.036 |
| RA2 LE EA | 0.702±0.016 | 0.155±0.012 | 0.232±0.040 | 0.106±0.039 |
| *Hyp 2D* | **0.787**±0.019 | **0.325**±0.036 | **0.510**±0.061 | **0.393**±0.067 |
| Gpcr | | | | |
| ORG LE OD | 0.681±0.006 | 0.251±0.011 | 0.874±0.039 | 0.827±0.059 |
| ORG LE EA | 0.790±0.007 | 0.138±0.013 | 0.378±0.053 | 0.217±0.050 |
| REV LE OD | 0.682±0.010 | 0.251±0.010 | **0.876**±0.037 | **0.832**±0.056 |
| REV LE EA | 0.790±0.008 | 0.137±0.011 | 0.344±0.042 | 0.181±0.038 |
| RA1 LE OD | 0.646±0.004 | 0.102±0.003 | 0.396±0.065 | 0.301±0.048 |
| RA1 LE EA | 0.790±0.006 | 0.138±0.007 | 0.296±0.051 | 0.169±0.036 |
| RA2 LE OD | 0.624±0.040 | 0.085±0.013 | 0.288±0.065 | 0.204±0.057 |
| RA2 LE EA | 0.792±0.004 | 0.138±0.006 | 0.282±0.038 | 0.149±0.029 |
| *Hyp 2D* | **0.839**±0.007 | **0.343**±0.020 | 0.702±0.093 | 0.607±0.106 |
| Ion | | | | |
| ORG LE OD | 0.858±0.001 | 0.425±0.007 | **0.954**±0.049 | **0.937**±0.058 |
| ORG LE EA | 0.859±0.003 | 0.362±0.006 | 0.836±0.028 | 0.715±0.030 |
| REV LE OD | 0.857±0.002 | 0.425±0.004 | 0.952±0.029 | 0.935±0.038 |
| REV LE EA | 0.858±0.002 | 0.361±0.004 | 0.864±0.040 | 0.745±0.058 |
| RA1 LE OD | 0.852±0.002 | 0.310±0.004 | 0.624±0.044 | 0.480±0.054 |
| RA1 LE EA | 0.861±0.003 | 0.339±0.008 | 0.786±0.031 | 0.686±0.048 |
| RA2 LE OD | 0.842±0.001 | 0.209±0.003 | 0.294±0.034 | 0.175±0.026 |
| RA2 LE EA | 0.828±0.003 | 0.171±0.006 | 0.236±0.032 | 0.079±0.013 |
| *Hyp 2D* | **0.897**±0.007 | **0.510**±0.024 | 0.812±0.091 | 0.720±0.117 |
| Enz | | | | |
| ORG LE OD | 0.804±0.001 | 0.304±0.002 | 0.970±0.019 | 0.960±0.031 |
| ORG LE EA | 0.877±0.003 | 0.219±0.004 | 0.884±0.077 | 0.835±0.101 |
| REV LE OD | 0.804±0.001 | 0.306±0.003 | **0.982**±0.015 | **0.975**±0.021 |
| REV LE EA | 0.878±0.001 | 0.221±0.006 | 0.870±0.041 | 0.815±0.069 |
| RA1 LE OD | 0.760±0.002 | 0.042±0.001 | 0.032±0.021 | 0.008±0.008 |
| RA1 LE EA | 0.878±0.000 | 0.205±0.016 | 0.826±0.071 | 0.760±0.092 |
| RA2 LE OD | 0.744±0.007 | 0.040±0.004 | 0.036±0.030 | 0.011±0.012 |
| RA2 LE EA | 0.877±0.001 | 0.111±0.001 | 0.074±0.034 | 0.024±0.024 |
| *Hyp 2D* | **0.884**±0.005 | **0.587**±0.018 | 0.920±0.060 | 0.942±0.028 |

Supplementary Table 8. Performance of the bare-bone hyperbolic matrix factorization (*Hyp* 2D) carried out in the 2-dimensional hyperbolic space and different variants of the 2D Coalescent Embedding (CE) algorithm in 10 rounds of 10-fold CV benchmark.

|  | AUC | AUPR | PREC10 | AP |
| --- | --- | --- | --- | --- |
| Nr | | | | |
| ORG LE OD | 0.514±0.014 | 0.158±0.009 | 0.285±0.017 | 0.198±0.013 |
| ORG LE EA | 0.717±0.016 | 0.181±0.010 | 0.309±0.019 | 0.226±0.019 |
| REV LE OD | 0.507±0.025 | 0.168±0.012 | 0.291±0.025 | 0.204±0.017 |
| REV LE EA | 0.732±0.015 | 0.188±0.014 | 0.302±0.017 | 0.235±0.024 |
| RA1 LE OD | 0.485±0.015 | 0.131±0.008 | 0.245±0.020 | 0.125±0.015 |
| RA1 LE EA | 0.725±0.016 | 0.170±0.010 | 0.273±0.026 | 0.169±0.014 |
| RA2 LE OD | 0.491±0.015 | 0.124±0.004 | 0.224±0.018 | 0.115±0.012 |
| RA2 LE EA | 0.723±0.015 | 0.168±0.008 | 0.266±0.040 | 0.137±0.029 |
| *Hyp 2D* | **0.826**±0.018 | **0.369**±0.020 | **0.496**±0.050 | **0.417**±0.063 |
| Gpcr | | | | |
| ORG LE OD | 0.678±0.004 | 0.257±0.005 | 0.764±0.027 | 0.710±0.028 |
| ORG LE EA | 0.793±0.002 | 0.142±0.005 | 0.332±0.020 | 0.174±0.020 |
| REV LE OD | 0.680±0.005 | 0.257±0.004 | **0.797**±0.028 | **0.727**±0.032 |
| REV LE EA | 0.795±0.004 | 0.142±0.005 | 0.330±0.022 | 0.185±0.028 |
| RA1 LE OD | 0.654±0.003 | 0.105±0.003 | 0.322±0.023 | 0.222±0.021 |
| RA1 LE EA | 0.795±0.002 | 0.139±0.003 | 0.297±0.041 | 0.149±0.029 |
| RA2 LE OD | 0.635±0.013 | 0.086±0.008 | 0.237±0.041 | 0.136±0.033 |
| RA2 LE EA | 0.795±0.003 | 0.141±0.002 | 0.244±0.018 | 0.126±0.010 |
| *Hyp 2D* | **0.864**±0.006 | **0.386**±0.011 | 0.763±0.074 | 0.648±0.117 |
| Ion | | | | |
| ORG LE OD | 0.860±0.001 | 0.429±0.002 | 0.935±0.022 | 0.912±0.025 |
| ORG LE EA | 0.860±0.002 | 0.358±0.003 | 0.832±0.011 | 0.721±0.017 |
| REV LE OD | 0.859±0.001 | 0.429±0.003 | **0.943**±0.028 | **0.920**±0.039 |
| REV LE EA | 0.860±0.002 | 0.358±0.006 | 0.816±0.030 | 0.721±0.037 |
| RA1 LE OD | 0.860±0.001 | 0.357±0.003 | 0.744±0.026 | 0.577±0.035 |
| RA1 LE EA | 0.861±0.001 | 0.338±0.005 | 0.773±0.024 | 0.662±0.028 |
| RA2 LE OD | 0.844±0.001 | 0.211±0.002 | 0.269±0.027 | 0.127±0.015 |
| RA2 LE EA | 0.829±0.001 | 0.173±0.003 | 0.254±0.020 | 0.104±0.016 |
| *Hyp 2D* | **0.919**±0.003 | **0.645**±0.008 | 0.913±0.006 | 0.860±0.021 |
| Enz | | | | |
| ORG LE OD | 0.804±0.001 | 0.299±0.001 | 0.965±0.007 | 0.947±0.013 |
| ORG LE EA | 0.878±0.000 | 0.200±0.002 | 0.708±0.047 | 0.621±0.054 |
| REV LE OD | 0.804±0.001 | 0.299±0.001 | 0.975±0.007 | 0.960±0.014 |
| REV LE EA | 0.878±0.001 | 0.203±0.005 | 0.735±0.042 | 0.644±0.054 |
| RA1 LE OD | 0.759±0.001 | 0.041±0.001 | 0.045±0.017 | 0.008±0.006 |
| RA1 LE EA | 0.879±0.001 | 0.199±0.003 | 0.682±0.037 | 0.594±0.052 |
| RA2 LE OD | 0.749±0.005 | 0.040±0.003 | 0.068±0.037 | 0.021±0.013 |
| RA2 LE EA | 0.878±0.001 | 0.112±0.001 | 0.083±0.029 | 0.030±0.016 |
| *Hyp 2D* | **0.914**±0.003 | **0.615**±0.014 | **0.994**±0.040 | **0.983**±0.045 |

|  |
| --- |
| **Supplementary Fig. 1.** The average time spent by different methods in predicting missing links in the Nuclear Receptor (Nr) test set: Cannistraci’s local topology methods (LOC TOP), Coalescent Embedding (CE), Euclidean Logistic Matrix Factorization (Euc), and Hyperbolic Factorization (Hyp). Each method was run with default parameters. The program for computing local topology (LOC TOP) measures, as implemented by the authors, returns multiple link weights, including CAR, CJC, CPA, CAA, and CRA. Hence, computing each measure can be made even faster than shown. |

**REFERENCES:**

1. [] Liu, Y., Wu, M., Miao, C., Zhao, P. & Li, X. Neighborhood regularized logistic matrix factorization for drug-target interaction prediction. *PLoS Computational Biology* 12, e1004760 (2016). [↑](#endnote-ref-1)
2. [] Hao, M., Bryant, S. H., & Wang, Y. (2017). Predicting drug-target interactions by dual-network integrated logistic matrix factorization. *Scientific Reports*, *7*(1), 1-11. [↑](#endnote-ref-2)
3. [] Wang, S., Li, J., Wang, Y., & Juan, L. (2021). A neighborhood-based global network model to predict drug-target interactions. *IEEE/ACM Transactions on Computational Biology and Bioinformatics*. [↑](#endnote-ref-3)
4. [] Ratcliffe, J. G., Axler, S., & Ribet, K. A. (2006). Foundations of hyperbolic manifolds (Vol. 149). New York: Springer. [↑](#endnote-ref-4)
5. [] Daminelli, S., Thomas, J. M., Durán, C., & Cannistraci, C. V. (2015). Common neighbours and the local-community-paradigm for topological link prediction in bipartite networks. *New Journal of Physics*, *17*(11), 113037. [↑](#endnote-ref-5)
